# Trm112, a Protein Activator of Methyltransferases Modifying Actors of the Eukaryotic Translational Apparatus

**DOI:** 10.3390/biom7010007

**Published:** 2017-01-27

**Authors:** Gabrielle Bourgeois, Juliette Létoquart, Nhan van Tran, Marc Graille

**Affiliations:** 1 Laboratoire de Biochimie, Ecole polytechnique, CNRS, Université Paris-Saclay, 91128 Palaiseau CEDEX, France; gabrielle.bourgeois@polytechnique.edu (G.B.); juliette.letoquart@uclouvain.be (J.L.); nhan.tran-van@polytechnique.edu (N.v.T.); 2 De Duve Institute, Université Catholique de Louvain, avenue Hippocrate 75, 1200 Brussels, Belgium

**Keywords:** post-transcriptional modification, RNA modifying enzyme, methyltransferase, *S*-adenosyl-l-methionine, translation

## Abstract

Post-transcriptional and post-translational modifications are very important for the control and optimal efficiency of messenger RNA (mRNA) translation. Among these, methylation is the most widespread modification, as it is found in all domains of life. These methyl groups can be grafted either on nucleic acids (transfer RNA (tRNA), ribosomal RNA (rRNA), mRNA, etc.) or on protein translation factors. This review focuses on Trm112, a small protein interacting with and activating at least four different eukaryotic methyltransferase (MTase) enzymes modifying factors involved in translation. The Trm112-Trm9 and Trm112-Trm11 complexes modify tRNAs, while the Trm112-Mtq2 complex targets translation termination factor eRF1, which is a tRNA mimic. The last complex formed between Trm112 and Bud23 proteins modifies 18S rRNA and participates in the 40S biogenesis pathway. In this review, we present the functions of these eukaryotic Trm112-MTase complexes, the molecular bases responsible for complex formation and substrate recognition, as well as their implications in human diseases. Moreover, as Trm112 orthologs are found in bacterial and archaeal genomes, the conservation of this Trm112 network beyond eukaryotic organisms is also discussed.

## 1. Introduction

Gene expression is a finely-tuned process allowing a cell to adapt rapidly throughout the cell cycle or upon exposure to environmental cues. Several layers of regulation are known, and among those, nucleic acid and protein modifications (phosphorylation, methylation, acetylation, etc.) are particularly important. It is indeed well known that DNA modifications and post-translational modifications of histone proteins participate in the epigenetic control of gene expression [1]. Similarly, the activity of factors involved in translation is enhanced or regulated by post-transcriptional or post-translational modifications with methylation being the most prominent one in protein translation. This is particularly the case for transfer RNAs (tRNA), which are heavily modified so as to improve their stability, as well as the efficiency and accuracy of translation [2]. Ribosomal RNAs’ (rRNA) maturation processes also include various post-transcriptional modifications, including 2’-OH methylation, base methylation, pseudo-uridylation or more complex modifications [3]. The emerging field of epitranscriptomics has shed light on several modified nucleotides on messenger RNAs (mRNA), such as *N*^6^-methyladenosine (m^6^A, [4,5]), *N*^1^-methyladenosine (m^1^A, [6]), pseudouridine [7] and 5-(hydroxy-)methylcytosine [8,9]. Finally, an increasing number of ribosomal proteins and of translational factors is also subject to post-translational modifications [10].

In this review, we present the current knowledge on the Trm112 eukaryotic protein, which acts as an activating platform of four *S*-adenosyl-l-methionine (SAM)-dependent methyltransferases (MTases) modifying rRNA (Bud23), tRNAs (Trm9 and Trm11) or the eRF1 class I translation termination factor (Mtq2), perfectly illustrating the importance of methylation in protein synthesis. Potential Trm112 prokaryotic orthologs are also discussed.

## 2. Eukaryotic Trm112 Network

### 2.1. Trm112 

Trm112, a 15-kDa protein, is widely conserved in eukaryotic organisms. It has been mostly characterized by studies first conducted in *Saccharomyces cerevisiae* (*Sc*) and later in human cells. In baker’s yeast, the *TRM112* gene was initially considered as an essential gene from a large-scale survey of the growth phenotype resulting from systematic deletion of individual genes [11]. However, further studies revealed that the *trm112∆* yeast strain is very sick, but still viable [12,13,14,15]. Studies performed on SMO2, the *Arabidopsis thaliana* Trm112 ortholog, have shown that as in *S. cerevisiae*, the inactivation of the *SMO2* gene leads to a defect in cell growth [16]. *SMO2* is also required for proper cell division and development, but the mechanisms underlying these phenotypes are still unknown. Finally, the mouse Trm112 ortholog is strongly and ubiquitously expressed during mouse embryo development [17].

**Figure 1:**
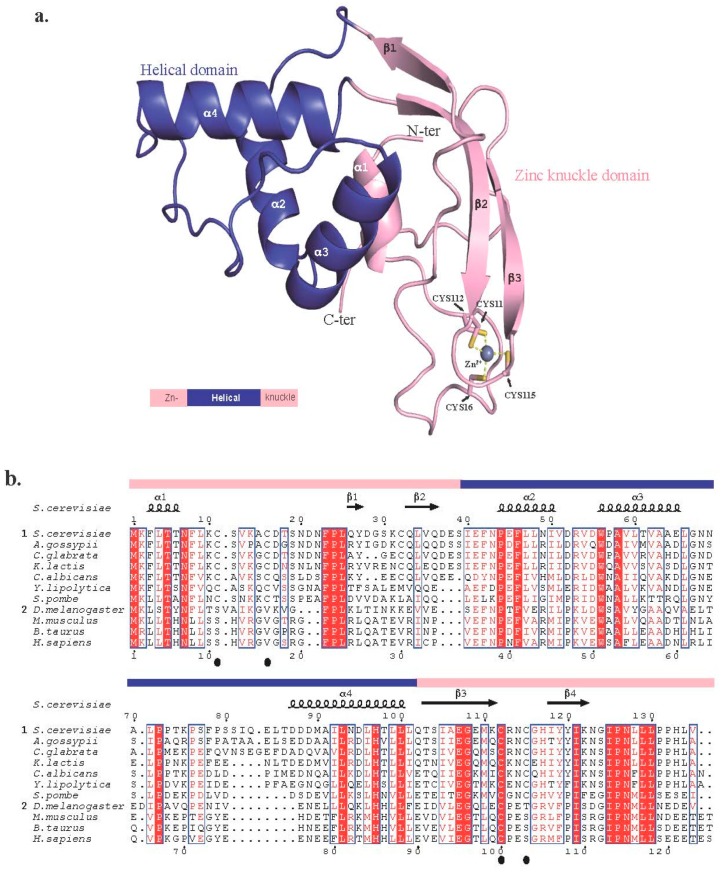
Organization of eukaryotic Trm112 proteins. (**a**) Ribbon representation of the crystal structure of isolated *Saccharomyces cerevisiae* Trm112 protein with a schematic representation of eukaryotic Trm112 shown below with the domain’s color code. (**b**) Sequence alignment of eukaryotic Trm112 protein sequences. Amino acids forming the Zn-knuckle and helical domains are identified by pink and blue bars, respectively, above the sequences. The positions of the four cysteine residues coordinating the zinc atom in the structures of fungal and *Encephalitozoon cuniculi* Trm112 proteins are indicated by black spheres below the alignment. Secondary structure elements as observed in the structure of the *S. cerevisiae* Bud23-Trm112 complex are indicated above the sequences [20]. Sequences have been divided into two subgroups: fungal proteins (Subgroup 1) and metazoans (Subgroup 2). Strictly-conserved residues are in white on a red background. Strongly-conserved residues are in red. This figure was generated using the Espript server [22]. C-ter: C-terminal protein extremity; N-ter: N-terminal protein extremity.

Sequence alignment of Trm112 orthologs from the three domains of life and crystal structures of eukaryotic Trm112 proteins either in an isolated form [18] or in complex with MTase partners (see below, [19,20,21]) have revealed an organization into two domains. The first domain, contributed by residues from the N- and C-terminal extremities of eukaryotic Trm112 proteins, is conserved within the three domains of life. It folds as a zinc-knuckle (Zn-knuckle) domain, composed of a short α-helix (α1) packed against the concave face of a curved anti-parallel β-sheet (Figure 1a). In the structure of isolated *Sc*Trm112 [18], this β-sheet is composed of three β-strands, and the Trm112 C-terminal extremity folds back onto a hydrophobic region of the Zn-knuckle domain. In the crystal structures of Trm112-MTase complexes [19,20,21], this Trm112 C-terminal extremity adopts a radically different conformation and folds as a fourth β-strand (β4), which is engaged in the interaction with the MTase partners (see Section 2.5). The second domain is contributed by residues from the central region of Trm112 eukaryotic proteins and is absent in bacterial, as well as in some archaeal orthologs. Depending on the solved structures, this later domain is formed by three or four α-helices (see Section 3).

All structures of eukaryotic Trm112 solved to date are from fungi (*S. cerevisiae* [18,20], *Yarrowia lipolytica* [21]) or from an intracellular parasite (*Encephalitozoon cuniculi* [19]), and they all exhibit one zinc atom coordinated by four cysteine residues in the so-called Zn-knuckle domain. These residues belong to two well-conserved motifs (CX_3-4_C and CX_2_C from the N- and C-terminal parts, respectively; where C is for cysteine and X is for any amino acid; Figure 1b). However, these four cysteine residues are not conserved in metazoan Trm112 proteins, suggesting that Trm112 does not bind zinc in these organisms. A similar conservation scheme has been already observed for Ski2 helicase, a component of the SKI complex involved in the 3’ to 5’ mRNA decay in eukaryotic organisms. Indeed, fungal Ski2 orthologs harbor a zinc binding site formed by four conserved cysteine residues, while in metazoan Ski2 proteins, these residues are not conserved, but residues present at the corresponding positions may play the same structural role [23].

### 2.2. Role of Trm112 in tRNA Modification

#### 2.2.1. Trm11-Trm112 

**Figure 2:**
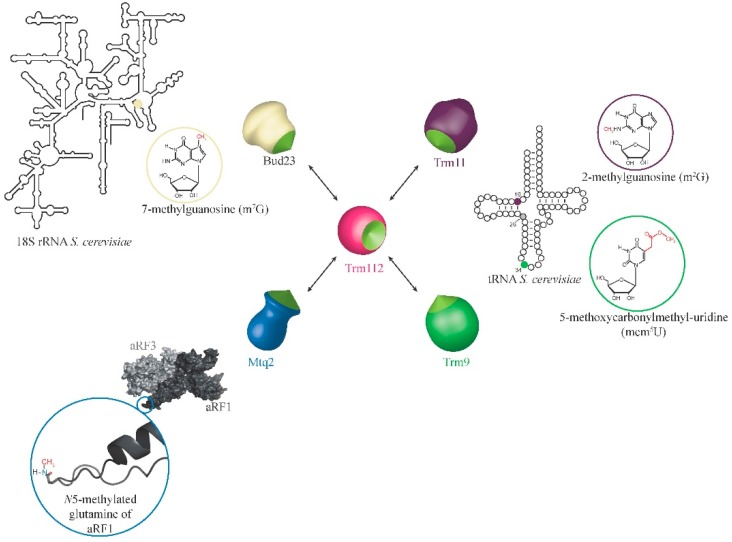
Schematic representation of the Trm112- methyltransferase (MTase) interaction network and of the substrates of these complexes. The surface representation of the aRF1-aRF3 complex from *Aeropyrum pernix* archeon was generated using PDB Code 3VMF [25]. Positions 10, 26 and 34 on a tRNA molecule are shown in purple, grey and green, respectively. The position of G_1575_ on 18S rRNA is shown as a beige sphere. The color code used to depict the various partners will be used in all figures of this review.

The first Trm112 partner that has been described is the tRNA MTase Trm11, which catalyzes the formation of *N*^2^-methylguanosine (m^2^G) at position 10 of some tRNAs [12]. This modification is conserved in archaea and eukaryotes, but absent in bacteria [24]. The m^2^G_10_ is part of the body of the tRNA and is likely involved in tRNA folding and stability. The m^2^G_10_ is stacked onto the m^2^_2_G_26_ nucleotide (Figure 2), which is methylated by Trm1. Interestingly, the simultaneous loss of both modifications induces strong growth defects ([12]). 

Bioinformatics analyses of eukaryotic Trm11 sequences suggested the presence of two domains. An N-terminal THUMP domain (for thiouridine synthases, RNA methyltransferases and pseudouridine synthetases; [26]) formed by an NFLD (N-terminal ferredoxin-like domain) subdomain fused to a core-THUMP subdomain and a C-terminal class I SAM-dependent MTase domain [12,27]. Such modular organization has been confirmed by the recent crystal structure of the archaeal Trm11 ortholog from *Thermococcus kodakarensis* [28] and is shared with archaeal Trm14 and bacterial TrmN, which are both responsible for m^2^G formation at position 6 on tRNAs [29,30]. In the 4-thiouridine synthase enzyme ThiI, the THUMP domain was shown to interact with the 3’ CCA end [31] and was then proposed to position the substrate nucleotide in the enzyme active site. It would then act as a molecular ruler that controls the distance between the tRNA CCA end and the nucleotide to be modified.

In *S. cerevisiae*, Trm112 is needed for the formation of m^2^G_10_ modification by Trm11 [12], whereas archaeal orthologs studied so far (PAB1283 from *Pyrococcus abyssi* and aTrm11 from *Thermococcus kodakarensis*) are active on their own [28,32]. Initially, the m^2^G_10_ modification could only be recapitulated in vitro using the *Sc*Trm11-Trm112 complex either purified directly from yeast cells [12] or produced using a wheat germ cell-free translation system [33], suggesting that post-translational modifications might be necessary for enzymatic activity. More recently, the *Sc*Trm11-Trm112 complex purified following co-expression of both subunits in *Escherichia coli* turned out to be active on an in vitro synthesized tRNA. As mass spectrometry analyses showed that none of these proteins were post-translationally modified, post-translational/transcriptional modifications are then not mandatory for enzymatic activity [34]. Detailed analyses also indicate that Trm112 contributes to tRNA modification activity by influencing both SAM and tRNA binding either directly or indirectly [34].

Although studies on Trm11 exclusively focused on *S. cerevisiae* and archaeal proteins so far, an orthologous gene was identified in human genome through BLAST searches. Indeed, the product of the *C6orf75* gene is annotated as TRMT11, shares 34% identity and 54% similarity at the amino acid sequence level with Trm11, respectively, and has the same modular architecture as yeast Trm11. Two studies have linked defaults in the human TRMT11 gene or transcript with advanced prostate cancer [35,36], and this protein was shown to interact with at least three proteins from the p53-family in fruit-fly [37]. We have successfully purified the human TRMT11-TRMT112 complex following co-expression in *E. coli*, indicating that similarly to yeast Trm11, its human counterpart interacts directly with TRMT112. Future studies are now needed to characterize the biochemical and biological functions of this human complex.

#### 2.2.2. Trm9-Trm112 

The large-scale purification of budding yeast complexes using TAP-tag purification (tandem affinity purification) highlighted several partners for Trm112, including Trm9 [38]. The Trm9-Trm112 complex was further shown to be a tRNA MTase involved in the formation of mcm^5^(s^2^)U (5-methoxycarbonylmethyl(2-thio)uridine) modifications at position 34 from the anticodon loop of some tRNAs (Figure 2; [13,39]).

In *S. cerevisiae*, the formation of mcm^5^(s^2^)U involves at least 15 proteins. The first reaction, consisting of the addition of the carboxymethyl group at position 5 of the uracil to form 5-carboxymethyluridine (cm^5^U), is catalyzed by the Elongator complex (Elp1-Elp6), the activity of which is regulated by seven additional proteins [40,41,42]. During the second step, the Trm9-Trm112 complex methylates cm^5^U to yield mcm^5^U. This modification is present at the wobble position of tRNA^Arg^_(UCU)_, tRNA^Gly^_(UCC)_, tRNA^Lys^_(UUU)_, tRNA^Gln^_(UUG)_ and tRNA^Glu^_(UUC)_. In the last three tRNAs, the oxygen atom attached to the C2 atom of the uracil ring is further substituted by a sulfur atom via the Ncs6/Urm1 synthesis pathway to form mcm^5^(s^2^)U [43]. The presence of the methyl group is important for an efficient thiolation, as we detected tRNA mcm^5^U_34_ product, but not mcm^5^(s^2^)U_34_ after in vitro enzymatic reaction of *Sc*Trm9-Trm112 on tRNAs purified from the *trm9∆* yeast strain. Furthermore, several groups observed a drop in cm^5^(s^2^)U_34_ formation upon disruption of *TRM9* or *TRM112* genes [13,14,21]. In *S. cerevisiae*, this mcm^5^(s^2^)U_34_ tRNA modification confers susceptibility to zymocin, a toxin secreted by the yeast *Kluyveromyces lactis*, which cleaves specifically the modified anticodon loop, thereby inhibiting translation and leading to death [44]. 

The modifications in the anticodon loop of tRNAs are known to influence the translation rate and fidelity of decoding. Indeed, the mcm^5^(s^2^)U_34_ of tRNAs is involved in accurate and efficient reading of some codons in *S. cerevisiae* [45]. Two studies based on integrated analysis of proteome, transcriptome and ribosome foot-printing highlighted the link between these tRNA modifications and the regulation of global protein expression [45,46]. The lack of mcm^5^(s^2^)U_34_ modifications, upon deletion of the *TRM9* gene, results in an increase of ribosomal pausing on mRNAs enriched with AGA and GAA codons. It is noteworthy that a significant portion of these mRNAs encodes proteins involved in protein synthesis, cell cycle control or DNA damage response, and consequently, those proteins undergo a decreased expression in the *trm9∆* strain. These results rationalize the sensitive phenotype of the *trm9∆* strain to methyl methanesulfonate (MMS) exposure. Indeed, MMS is a DNA damaging agent that methylates DNA mainly on the *N*^7^ and *N*^3^ atoms of G and A bases, respectively, but also at other oxygen and nitrogen atoms of DNA bases and, thereby, triggers DNA repair machineries. The *trm9∆* strain also presents a delay in transition from the G1 to the S phase upon exposure to MMS [45]. Likewise, the absence of Trm9-catalyzed methylation causes translational infidelity and activation of protein stress response pathways [47].

**Figure 3:**
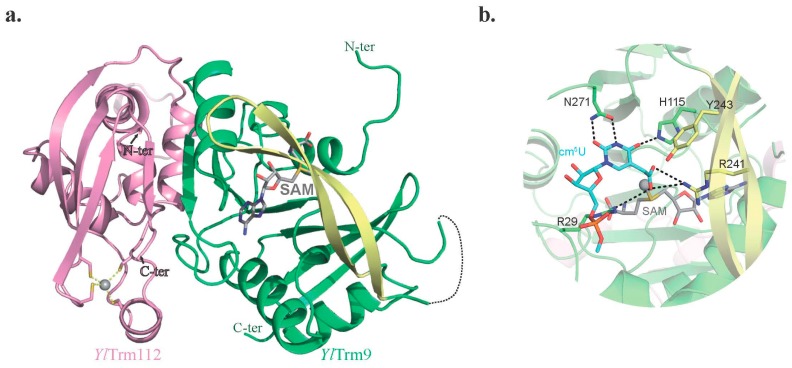
Crystal structure of the *Yarrowia lipolytica* (*Yl*) Trm9-Trm112 complex. (**a**) Ribbon representation of the *Yl*Trm9-Trm112 complex. The *S*-adenosyl-l-methionine (SAM) molecule (grey sticks), which was absent in the crystal structure, has been modeled by superimposing the SAM-bound structure of Bud23 onto *Yl*Trm9. The Trm9 lid is colored yellow. (**b**) Model of cm^5^U_34_ (blue sticks) docked into the *Yl*Trm9 active site. The SAM methyl group to be transferred is depicted as a sphere. Residues are numbered according to the *S. cerevisiae* protein.

To obtain information on the Trm9 active site and Trm9-Trm112 complex organization, we solved the X-ray structure of this complex from the yeast *Yarrowia lipolytica* (*Yl*; [21]). *Yl*Trm9 adopts the classical class I SAM-dependent MTase fold with a central seven-stranded β-sheet surrounded by two α-helices on each side. Moreover, a twisted two-stranded β-sheet forms a lid located on top of the C-terminal extremity of the central β-sheet and projects onto the active site (Figure 3a). Based on this structure, we mapped the active site of the *Sc*Trm9-Trm112 complex. This led to the identification of several mutants (R29A, H115A, R241A, Y243A and N271A) that strongly affect the affinity for tRNA (but not for SAM), as well as the enzymatic activity (k_cat_). This supports a role of these conserved residues in tRNA binding and particularly in the optimal orientation of the cm^5^U_34_ nucleotide substrate in the active site, which is required for an efficient methyl transfer reaction by the SN_2_ mechanism (Figure 3b; [21]).

Trm9 is largely conserved in eukaryotes. In human, two Trm9 orthologs are present: ABH8 and hTrm9L. ABH8 is a bifunctional enzyme encompassing a Trm9-like MTase domain converting cm^5^U into mcm^5^U, fused to an RRM domain (for RNA-recognition motif) and an AlkB-like (alpha-ketoglutarate-dependent dioxygenase) domain responsible for the hydroxylation of mcm^5^U into (S)-mchm^5^U ((S)-5-methoxycarbonylhydroxymethyluridine; [48,49,50]). Similarly to yeast, ABH8 needs to interact with TRMT112 to be active. The ABH8 protein is highly expressed in a variety of human cancer cells, such as bladder cancer cells, and its silencing suppresses tumor growth, angiogenesis and metastasis by inducing the apoptosis of urothelial carcinoma cells [51]. ABH8 depletion also renders cells sensitive to DNA damaging agents (MMS) and to the bleomycin anti-cancer drug [48]. Compared to ABH8, hTrm9L is only made of the MTase domain. The hTrm9L protein presents a Trm9-like tRNA MTase activity, but to our knowledge, its interaction with TRMT112 has not been characterized [52]. It acts as a negative regulator of tumor growth, and the tumor cells deleted for the gene encoding hTrm9L are sensitive to paromomycin and gentamycin antibiotics [52]. In *A. thaliana* (*At*), AT1G31600 (*At*TRM9), which is similar to Trm9 MTase, catalyzes the formation of mcm^5^U_34_, and its activity is dependent on two Trm112 orthologs (*At*TRM112a and *At*TRM112b; [53]). A second protein AT1G36310 (*At*ALKBH8), with similarity to ABH8 RRM and AlkB-like domains, has been shown to catalyze the hydroxylation of mcm^5^U into (S)-mchm^5^U [53]. 

### 2.3. Role of Trm112 in Translation Termination: Mtq2-Trm112

Translation termination occurs when a stop codon is present in the ribosomal A-site. It is then not recognized by a cognate tRNA, but by a protein factor known as class I release factor (RF1 or RF2 in bacteria, eRF1 in eukaryotes and aRF1 in archaea), which triggers the release of the newly-synthesized proteins. Class I release factors are tRNA mimics, as they recognize the stop codon in the A-site through one domain and project a universally-conserved GGQ motif (for Gly-Gly-Gln) from another domain into the ribosomal peptidyl transferase center [54,55]. In bacteria, the side chain of the glutamine residue from this motif is *N*^5^-methylated by the PrmC MTase (also known as HemK). This post-translational modification is important for normal translation termination in vivo and increases the affinity of the release factor for ribosomes [56,57,58,59,60,61]. Interestingly, the glutamine side chain of the GGQ motif from the eukaryotic class I release factor is also *N*^5^-methylated (Figure 2; [62,63]). The enzyme responsible for this modification in *S. cerevisiae* yeast is the Mtq2-Trm112 complex, where Mtq2 is the MTase catalytic subunit. Furthermore, this enzyme modifies eRF1 only when this latter one is associated with the GTP-bound form of class II translation termination factor eRF3 [18,62]. Mtq2 orthologs have been described in human (HEMK2) and mouse (PRED28), where they also form a complex with the corresponding Trm112 orthologs and modify eRF1 translation termination factor [64]. 

**Figure 4:**
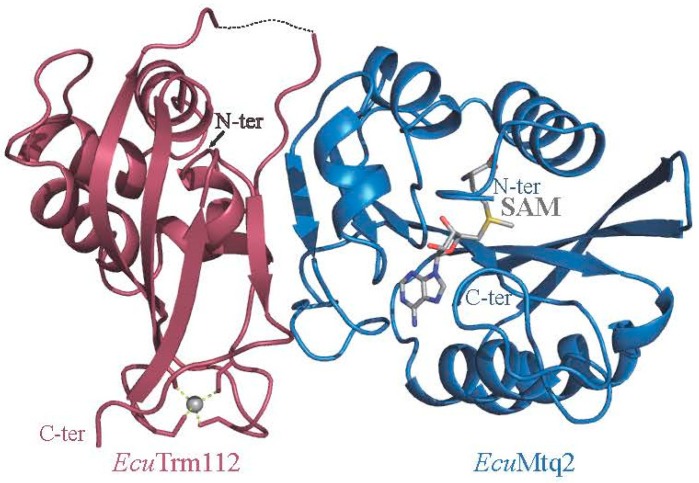
Ribbon representation of the crystal structure of the *Encephalitozoon cuniculi* (*Ecu*) Mtq2-Trm112 complex bound to SAM (grey sticks).

The crystal structure of Mtq2-Trm112 complex from *Encephalitozoon cuniculi* (*Ecu*) parasite obtained in the presence of SAM bound to the Mtq2 catalytic subunit has confirmed the prediction that Mtq2 is a class I SAM-dependent MTase (Figure 4). It has also revealed the presence of a highly conserved surface surrounding the SAM methyl group [19]. This region displays a negatively-charged potential, which can ideally interact with the numerous positively-charged and conserved residues surrounding the eRF1 GGQ motif. Furthermore, the crystal structure of the GTP-bound form of the archaeal aRF1-aRF3 complex (orthologous to the eukaryotic eRF1-eRF3 complex) reveals that aRF3 switches I and II regions, which are known to adopt different conformations between the GDP- or GTP-bound forms, are in close proximity of the GGQ motif [25]. Hence, these switches’ regions are very likely to interact directly with the Mtq2-Trm112 complex, thereby explaining its specificity for the eRF1-eRF3-GTP form [18]. Comparison of the crystal structures of *Ecu*Mtq2-Trm112 and *E. coli* PrmC-RF1 complexes reveals that the NPPY (for Asn-Pro-Pro-Tyr) active site signature from PrmC, which coordinates the RF1 GGQ motif for proper methylation, structurally matches with the Mtq2 NPPY signature, supporting a similar recognition mode of the GGQ motif across the domains of life. Finally, it has also been shown that in the absence of TRMT112, HEMK2 does not exhibit enzymatic activity and cannot bind SAM, while the purified HEMK2-TRMT112 complex is active and binds SAM. This indicates that TRMT112 activates HEMK2 by stimulating SAM binding [19,64].

To date, the role of eRF1 methylation remains obscure, but the conservation of this post-translational modification on the GGQ motif of at least bacterial and eukaryotic class I release factors, which adopt radically different three-dimensional structures, argues in favor of an important functional role. This is further supported by the growth defect phenotype of yeast cells lacking the *MTQ2* gene [13,14,63], the cell proliferation defect with arrest in the G1 phase of murine embryonic stem cells depleted of the PRED28α isoform [65], the early mouse embryonic lethality upon disruption of PRED28α isoform [66] and the two-fold reduction in HEK293 human cells growth rate resulting from stable knock-down of the *HEMK2* gene [66]. Finally, murine PRED28α and human HEMK2 proteins appear to have a broad substrate specificity [67]. Hence, future studies aimed at clarifying the role of the eukaryotic Mtq2-Trm112 complexes and of the methylation they are catalyzing are needed.

### 2.4. Role of Trm112 in Ribosome Biogenesis

The deletion of the *TRM112* gene in *S. cerevisiae* results in a strong growth defect phenotype associated with strong defects in the synthesis of both ribosomal subunits and an increased sensitivity to paromomycin, a well-known inhibitor of protein synthesis [15,68]. Recent studies have started to decipher Trm112’s role in the very complex process of ribosome biogenesis.

#### 2.4.1. The Bud23-Trm112 Complex Is Involved in 40S Maturation

To shed light on Trm112’s role in 40S ribosomal subunit synthesis, a TAP-tag purification was conducted under milder conditions than in the initial large-scale study performed by Gavin et al. [38], and new potential partners were identified by mass spectrometry analysis. Among these, Bud23, a SAM-dependent MTase involved in ribosome biogenesis and catalyzing the methylation of N^7^ atom of G_1575_ in 18S rRNA, was an attractive candidate (Figure 2; [15,69]). The interaction between Bud23 and Trm112 was shown to be direct by co-purification of both proteins following co-expression in *E. coli* [15,68]. Furthermore, Trm112 is important for Bud23 cellular stability and for its activity [15]. In *S. cerevisiae*, the *bud23∆* strain exhibits a strong growth defect, sensitivity to paromomycin, as well as defects in the synthesis and the nuclear export of the small ribosomal subunit 40S [15,69]. This *bud23∆* mutant is affected in A2 cleavage, resulting in an accumulation of 35S and 20S rRNA intermediates, a depletion of 27SA2 rRNA intermediate and consequently a reduction of mature 18S rRNA [15,69]. Finally, Bud23 associates with the 90S particle at the intermediate stage before A2 cleavage [70].

**Figure 5:**
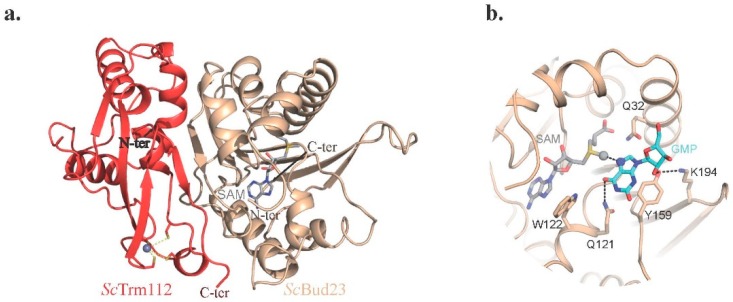
Crystal structure of the *S. cerevisiae* (*Sc*) Bud23-Trm112 complex. (**a**) Ribbon representation of the *Sc*Bud23-Trm112 complex bound to SAM. (**b**) Model of guanosine monophosphate (GMP) (blue sticks) bound to the *Sc*Bud23 active site.

Crystal structures of the *Sc*Bud23-Trm112 complex (lacking the Bud23 C-terminal extension rich in basic residues) in the presence or absence of SAM have brought useful information regarding the interaction mode between both proteins (see Section 2.5), but also on G_1575_ binding (Figure 5; [20]). Indeed, comparison of the crystal structures of *Sc*Bud23 and *Coffea canephora* xanthine MTase bound to xanthosine has revealed striking similarities between enzyme active sites, suggesting that they both bind the purine ring of their substrates in a very similar manner [20,71]. This binding mode was validated experimentally by the characterization of Bud23 active site mutants. Based on these observations, the *Sc*Bud23-Trm112-GMP (guanosine monophosphate) model was generated (Figure 5B) and superimposed onto nucleotide G_1575_ in the structure of the mature *S. cerevisiae* 80S ribosome. Such superimposition reveals large steric clashes between Bud23-Trm112 and ribosomal components, indicating that this Bud23-Trm112 complex cannot bind mature ribosomes and has to dissociate from the 40S subunit before completion of its biosynthesis. Additional experiments demonstrated that although Bud23 is recruited to pre-ribosomes at an early nucleolar stage, G_1575_ methylation, which is not essential for Bud23 cellular function, is a late event, as it occurs on the 20S pre-rRNA [20]. Finally, the Bud23-Trm112 complex physically interacts with the Dhr1 DEAH-helicase (for Asp-Glu-Ala-His), which is involved in the dissociation of U3 small nucleolar RNA from the pre-40S prior to the formation of the central pseudo-knot of the 40S subunit [20,72,73]. 

The Bud23-Trm112 complex is also found in human cells where TRMT112 interacts with the human Bud23 ortholog RNMT2 (also known as WBSCR22/Merm1; [74,75]). Similarly to *Sc*Bud23, the RNMT2 protein, but not its MTase activity, is required for ribosome biogenesis [75,76]. RNMT2 is associated with several human diseases, as it is one of the several genes deleted in the Williams–Beuren neurodevelopmental syndrome [77,78]. It was also reported as a tumoral marker for invasive breast cancer, myeloma cells and hepatocarcinoma [79,80,81], and it might be involved in lung pathologies [82].

#### 2.4.2. Trm112 also Influences 60S Formation

The importance of Trm112 in ribosome biogenesis extends beyond the role of the Bud23-Trm112 complex in 40S maturation as the disruption of the *TRM112* gene in yeast also causes lower levels of the 60S subunit [15,68]. The effect of Trm112 depletion is less pronounced on 60S than on 40S levels, but Trm112 is definitely important for the synthesis of both subunits. Trm112’s role in 60S synthesis is supported by its co-immunoprecipitation with pre-60S and its association with Nop2 and Rcm1, two 25S rRNA-MTases involved in 60S biogenesis [68,83,84]. However, experimental evidence supporting a direct interaction between Trm112 and these two MTases has not been presented so far. Hence, Trm112’s role in 60S synthesis is still unclear, and future studies addressing this issue are needed. 

### 2.5. Common Themes in Recognition and Activation of These MTases by Trm112

Despite its small size, eukaryotic Trm112 is part of at least four heterodimeric MTase holoenzymes and acts as an activator of the MTase catalytic subunits. The description of the molecular mechanisms underlying Trm112’s activation role was hindered by the difficulty to express and purify most isolated MTase subunits (Trm9, Mtq2 and Bud23) in sufficient amount for biochemical and biophysical studies [13,15,18,68]. Indeed, only the co-expression of Trm112 together with each of these three MTases allows the purification of the corresponding Trm112-MTase complexes [13,14,15,18,19,21,64,68]. This first led to the determination of the crystal structures of three Trm112-MTase complexes, namely *Ecu*Mtq2-Trm112 [19], *Sc*Bud23-Trm112 [20] and *Yl*Trm9-Trm112 [21]. These structures revealed that Trm112 interacts in a very similar way with these three MTase partners (root mean square deviation (rmsd) values lower than 3 Å when superimposing the structures of the complexes). To date, no crystal structure of a Trm11-Trm112 complex has been determined. However, information on this complex could be gleaned from the possibility to purify *S. cerevisiae* Trm11 alone in milligram amounts [34]. Hydrogen-deuterium exchange experiments coupled to mass spectrometry revealed that Trm11 regions involved in Trm112 binding match with the regions from Trm9, Mtq2 and Bud23 MTase domains that interact with Trm112. Reciprocally, the Trm112 region contacted by Trm11 perfectly overlaps with the region involved in the interaction with the other MTases. Hence, these four MTases use the same surface of their MTase domain to bind to the same region of Trm112 and then compete directly to interact with Trm112. This is in agreement with previous reports showing that Trm11 over-expression in yeast reduces the amount of Trm112 co-immunoprecipitated with Trm9 and that Mtq2 over-expression in yeast results in decreased levels of Bud23 [15,85].

**Figure 6:**
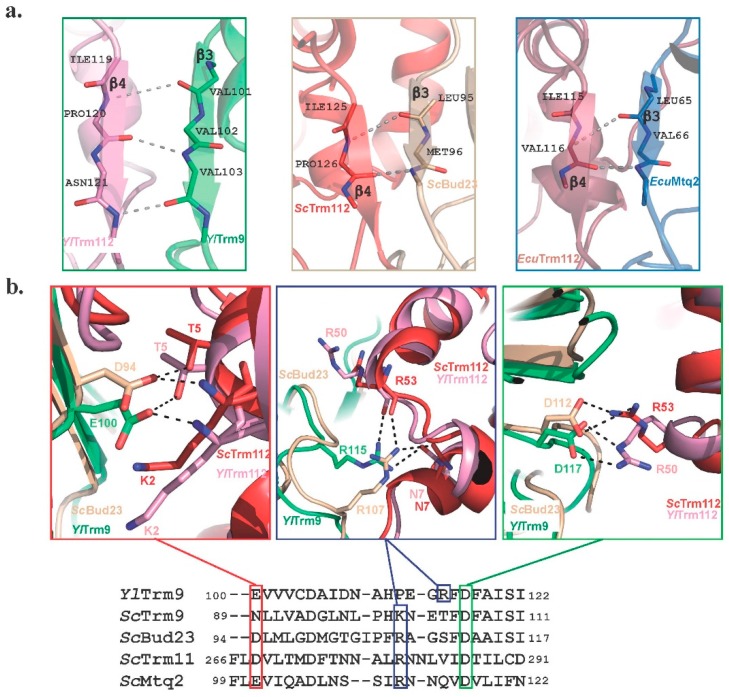
Comparison of the Trm112-MTase interfaces. (**a**) Ribbon representation of the β-zipper interaction between Trm112 and MTases. Hydrogen bonds formed between main chain atoms from both partners are depicted by grey dashed lines. (**b**) Comparison of *Sc*Bud23-Trm112 and *Yl*Trm9-Trm112 structures reveals conserved hotspots involved in complex formation. A structure-based sequence alignment of *Yl*Trm9 and of the four *S. cerevisiae* MTases interacting with Trm112 is shown in the lower panel. Only a small region of these MTases is shown for the sake of clarity.

These MTases interact mainly with the Zn-knuckle domain from Trm112, and complex formation buries a large hydrophobic region on the surface of both partners. This explains the requirement of Trm112 to express and purify three of these MTases in their soluble forms in *E. coli* and also to stabilize at least Bud23 in *S. cerevisiae* yeast cells [13,14,15,18,68]. Central to the Trm112-MTase interfaces is a β-zipper interaction formed between strand β3 from the MTase domain and strand β4 from Trm112, forming a continuous large eleven-stranded β-sheet (Figure 6a). Such interaction relies on hydrogen bonds formed between main chain atoms from both partners and hence is much more dependent on the local three-dimensional structure than on conservation of amino acid residues at the positions involved in the formation of this β-zipper. Finally, three electrostatic interactions are also observed in both fungal Trm9-Trm112 and Bud23-Trm112 complexes. Structure-based sequence alignment between these four MTases from the same organism shows that the residues present at each of these three positions are strictly conserved or have the same propensity to form similar electrostatic interactions, i.e., basic residues (Lys or Arg) or similar hydrogen bonding properties (Asp, Glu or Asn; Figure 6b). This observation indicates that these interactions are most likely common to all Trm112-MTase complexes. Altogether, such binding mode features explain that Trm112 can interact with several MTases sharing less than 20% sequence identity. The ability of plant and metazoan Trm112 or MTases orthologs to complement at least partially for the deletion of the corresponding yeast gene further supports the plasticity in the interaction mode between these proteins [21,52,64,76]. Indeed, this indicates that chimeric Trm112-MTase complexes can be formed between a yeast protein and an ortholog from its binding partner.

The activation role of Trm112 is not only restricted to its role in the stabilization of these catalytic MTase subunits in vivo. Indeed, it is also required for SAM binding. While in the absence of Trm112, well-folded *S. cerevisiae* Trm11 and human HEMK2 proteins do not bind SAM, these proteins bind SAM with micromolar affinity when in complex with Trm112 or TRMT112, respectively [19,34]. According to the SAM-bound structures of Mtq2-Trm112 and Bud23-Trm112 complexes, this property most likely results from the interaction between Trm112 and a loop from the MTase domain, which in turn directly interacts with the SAM molecule. Furthermore, Trm112 might also be important for substrate binding by at least Mtq2-Trm112 and Trm11-Trm112 complexes, as mutants located on the surface of Trm112 Zn-knuckle domain affect the enzymatic activity of these complexes without interfering with complex formation or SAM binding [19,34].

## 3. Trm112 in Prokaryotes

**Figure 7:**
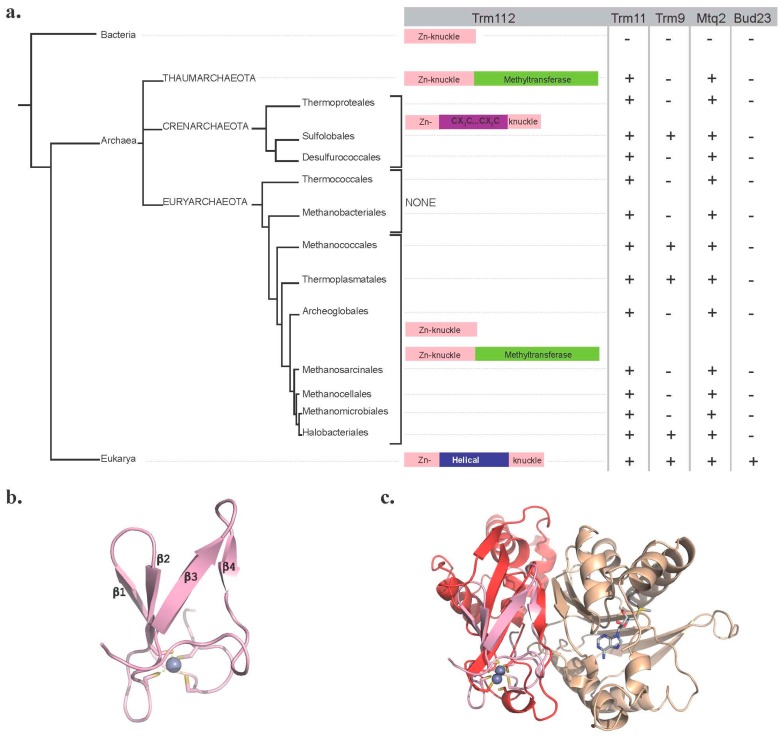
Trm112 is present in the three domains of life. (**a**) Simplified phylogenetic tree of Trm112. Emphasis is given to archaeal phylogeny. The distribution of Trm11, Trm9, Mtq2 and Bud23 proteins within the three domains of life is indicated. The various Trm112 forms identified are schematically depicted. (**b**) Ribbon representation of *Streptomyces coelicolor* SCO3027 protein nuclear magnetic resonance (NMR) structure (PDB Code 2KPI). Cysteine residues coordinating the zinc atom (grey sphere) bound to the protein are shown as sticks. (**c**) Superimposition of *Streptomyces coelicolor* SCO3027 structure (pink) onto Trm112 in the *Sc*Bud23-Trm112 complex (Trm112 and Bud23 are colored red and beige, respectively). The SAM molecule bound to Bud23 is shown as grey sticks.

Initial bioinformatics analyses have revealed the existence of proteins sharing sequence homology with yeast Trm112 within the three domains of life, with molecular weights ranging from 6 kDa in bacteria and archaea to around 15 kDa in eukaryotes (Figure 7a; [12,18]).

In bacteria, Trm112 orthologs are typically quite short (usual length around 60 residues), and the representative member from this family is YcaR from *E. coli*. According to the few NMR (nuclear magnetic resonance) structures determined by a structural genomics consortium (PDB Codes 2KPI and 2JS4; Figure 7b), these orthologs contain only a Zn-knuckle domain, which is highly similar to the corresponding domain in eukaryotic Trm112 proteins (rmsd value of 1.7–1.8 Å; Figure 7c). In these proteins, one zinc atom is coordinated by conserved cysteine and/or aspartic acid residues. To our knowledge, nothing is known about the function of bacterial Trm112 orthologs. No genes with significant sequence similarities with eukaryotic MTases known to interact with Trm112 could be detected in bacterial genomes. Furthermore, G_10_ on bacterial tRNAs is not methylated [24], and the wobble uridine (U_34_) from tRNAs’ reading codons ending with a purine harbors a mnm^5^U (5-methylaminomethyl-uridine) modification catalyzed by the MnmE-MnmG complex and the MnmC bifunctional enzyme [86]. As indicated in Section 2.3, the bacterial class I translation termination factors RF1 and RF2 are methylated on the glutamine side chain of their GGQ motif by the PrmC MTase, which is active on its own [58,59,60]. Finally, the only known m^7^G nucleotide found in 16S rRNA is located at position 527 in *E. coli* [87], a position differing radically from the one in eukaryotic 18S rRNA (G_1575_), and this modification is catalyzed by the RsmG MTase [88]. Hence, bacterial Trm112 are strongly conserved proteins with still unknown function. However, in some bacteria, Trm112 is fused to MTase domains, suggesting that similarly to eukaryotic Trm112, bacterial orthologs could interact with MTases. This is further supported by the strong structural similarity between bacterial and eukaryotic Trm112, in particular in the region involved in the interaction with MTases (Figure 7c). Future studies aimed at clarifying the role and the potential partners of these bacterial proteins are definitely needed.

Since the initial analyses conducted on archaeal Trm112 sequences [12,18], many more archaeal genomes have been sequenced, and the archaeal phylogeny has been revised [89]. A new sequence analysis showed that Trm112 archaeal orthologs cluster into three subfamilies (Figure 7a). The first one composed of proteins ranging in size from 60–80 residues harbors a [C/D]PX[C/D]X_19-36_CX_2_C signature (where C, D, P and X are for cysteine, aspartic acid, proline and any residue, respectively). It is predicted to contain only the Zn-knuckle domain similarly to bacterial orthologs and is found almost exclusively in Euryarchaeota. The second corresponds to proteins of about 130–140 amino acids found exclusively in the Crenarchaeota phylum. These proteins contain the Zn-knuckle domain, as well as a central region. This latter one displays some similarity with eukaryotic Trm112 helical domain and a conserved putative CX_3_CX_15-20_CX_2_C Zn-binding signature. This observation is compatible with the eocyte phylogenetic tree proposed by Cox et al. [90], proposing that Crenarchaeota are most closely related to eukaryotes. The third subfamily (composed of at least 29 members) is formed by proteins consisting of an N-terminal Trm112-like Zn-knuckle domain fused to a C-terminal SAM-dependent MTase domain. These are found only in some Euryarchaeota or Thaumarchaeota, but not in Crenarchaeota. Interestingly, bioinformatic analyses of the sequence of the MTase domains fused to Trm112 reveal that these MTases do not all belong to the same family and that some of these are putative archaeal orthologs of Trm9. Finally, no protein with significant sequence similarity with Trm112 could be identified in Thermococcales and Methanobacteriales from the Euryarchaeota phylum. This is noteworthy as Trm11 orthologs from two Thermococcales archaea (*Pyrococcus abyssi* and *Thermococcus kodakarensis*) can form m^2^G (and even m^2^_2_G) at position 10 of some tRNAs in vitro without the requirement of a protein partner [28,32]. Bioinformatics analysis supports the existence of Trm11 orthologs in all archaea phyla, and hence, most of the time, these proteins co-occur with Trm112, suggesting that with the exception of Thermococcales and Methanobacteriales Trm11, the other archaeal Trm11 orthologs may exist as a complex with Trm112 and may require Trm112 to be active as observed in eukaryotes. Similarly, Mtq2 orthologs are present in all archaea phyla. The presence of Mtq2 orthologs in archaea is fully consistent with the conservation of the GGQ signature on eukaryotic (eRF1) and archaeal (aRF1) class I translation termination factors and their strong structural similarity [25,91,92].

Modifications of archaeal tRNAs were investigated primarily in *Haloferax volcanii*, and U at position 34 of some tRNAs was shown to harbor an unknown modification [93]. A more recent study identified 5-carbamoylmethyluridine (ncm^5^U_34_) at position 34 from tRNA^Leu^(UAG) isolated from *Thermoplasma acidophilum* (Thermoplasmatales; Euryarchaeota; [94]). Several observations led Grosjean et al. to propose that mcm^5^(s^2^)U could also be present in *Haloferax volcanii* (Halobacteriales; Euryarchaeota) and *Sulfolobus solfataricus* (Sulfolobales; Crenarchaeota) [95]. Indeed, Elp3 (HVO_2888), Tuc1 (HVO_0580) and Trm9 (HVO_0574) orthologs were initially predicted in both organisms, and genes encoding putative Elp3 and Trm9 orthologs are clustered in *Sulfolobus solfataricus.* More recently, HVO_1032 was proposed as a new *H. volcanii* Trm9 ortholog due to a better blast score [96]. In addition, recombinant Elp3 from *Methanocaldococcus infernus* archeon (Methanococcales, Euryarchaeota) was shown to catalyze cm^5^U formation on tRNAs in vitro [97]. Blast analysis of the *H. volcanii* HVO_1032 protein sequence against archaeal proteins identified Trm9 putative orthologs with E-values lower than 1e^−48^ only in Sulfolobales (Crenarchaeota) and in Halobacteriales (Euryarchaeota), suggesting a limited distribution of this modification. Finally, no protein with significant sequence homology with Bud23 could be identified by bioinformatics searches. This is in agreement with the fact that to our knowledge, the nucleotide that structurally matches with *S. cerevisiae* 18S rRNA G_1575_ has not been shown to be modified in archaeal 16S rRNAs analyzed so far.

Many interesting common features exist between archaeal and eukaryotic Trm112 proteins, opening a large field of investigation for future research aimed at understanding the functions of Trm112 in archaea. Those studies could also contribute to improving our understanding of the role of Trm112 in eukaryotes based on these similarities.

## 4. Conclusions

Trm112 proteins are almost ubiquitously found in the three domains of life. Most of our knowledge on Trm112 function comes from studies performed in eukaryotic organisms, where Trm112 is known to interact with and activate at least four MTases modifying factors involved in various facets of translation and linked to human diseases. Evidence supports that in archaea, Trm112 may exhibit an interaction network partially similar to that in eukaryotes, but this remains to be experimentally established. Finally, although Trm112 is also found in bacteria, its function(s) is (are) obscure and definitely needs to be clarified in this domain of life.
